# Prevalence of colonization by methicillin-resistant *Staphylococcus aureus* ST398 in pigs and pig farm workers in an area of Catalonia, Spain

**DOI:** 10.1186/s12879-016-2050-9

**Published:** 2016-11-28

**Authors:** Esteban Reynaga, Marian Navarro, Anna Vilamala, Pere Roure, Manuel Quintana, Marian Garcia-Nuñez, Raül Figueras, Carmen Torres, Gianni Lucchetti, Miquel Sabrià

**Affiliations:** 1 Department of Internal Medicine, Hospital Universitari de Vic, Barcelona, Spain; 2 Department of Medicine, Universitat Autònoma de Barcelona, Barcelona, Spain; 3 Microbiology Department, Hospital Universitari de Vic, Barcelona, Spain; 4 Epidemiology Department, Hospital Universitari de Vic, Barcelona, Spain; 5 Epidemiology Department Hospital Vall d’Hebron, Barcelona, Spain; 6 Infectious Diseases Unit, Health Sciences Research Institute of the Germans Trias i Pujol Foundation, Badalona, Barcelona Spain; 7 CIBER de Enfermedades Respiratorias, Madrid, Spain; 8 Veterinary, Associació Tecnicosanitària del Porcí (ASSAPORC), Vic, Barcelona Spain; 9 Area de Bioquímica y Biología Molecular, Universidad de La Rioja, Logroño, Spain; 10 Infectious Diseases Department, Hospital Germans Trias i Pujol, Badalona, Barcelona Spain

**Keywords:** MRSA ST398, ST398, Pig farmer, Pig, Livestock associated MRSA

## Abstract

**Background:**

A livestock-associated clonal lineage (ST398) of methicillin-resistant *Staphylococcus aureus* (MRSA) has been identified causing colonization or infection in farm workers. The aim of the study was to analyze the prevalence of MRSA-ST398 colonization in pigs and in pig farmers in an area with a high pig population (Osona, Barcelona province, Catalonia, Spain).

**Methods:**

We performed a cross-sectional prevalence study in Osona (Catalonia, Spain), from June 2014 to June 2015. All pig farm workers from 83 farms were studied. Twenty of these farms were randomly selected for the study of both pigs and farmers: 9 fattening and 11 farrow-to-finish farms. All workers over the age of 18 who agreed to participate were included. Samples were analyzed to identify MRSA-ST398 and their *spa* type.

**Results:**

Eighty-one of the 140 pig farm workers analyzed (57.9% (95% IC: 50.0–66.4%)) were MRSA-positive, all of them ST398. The mean number of years worked on farms was 17.5 ± 12.6 (range:1–50), without significant differences between positive and negative MRSA results (*p* = 0.763). Over 75% of MRSA-ST398 carriers worked on farms with more than 1250 pigs (*p* < 0.001). At least one worker tested positive for MRSA-ST398 on all 20 selected pig farms. Ninety-two (46.0% (95% IC: 39.0–53.0%)) of the nasal swabs from 200 pigs from these 20 farms were MRSA-positive, with 50.5% of sows and 41.4% of fattening pigs (*p* = 0.198) giving MRSA-positive results. All the isolates were tetracycline-resistant, and were identified as MRSA-ST398. The *spa* type identified most frequently was t011 (62%). Similar *spa* types and phenotypes of antibiotic resistance were identified in pigs and farmers of 19/20 tested farms.

**Conclusions:**

The prevalence of MRSA-ST398 among pig farm workers and pigs on farms in the studied region is very high, and the size of the farm seems to correlate with the frequency of colonization of farmers. The similar *spa*-types and phenotypes of resistance detected in pigs and workers in most of the farms studied suggest animal-to-human transmission.

## Background

Methicillin-resistant *Staphylococcus aureus* (MRSA) colonization can be acquired in the community (CA-MRSA) or in a hospital setting (HA-MRSA) [1]. Infections caused by CA-MRSA strains have been described, in some cases in individuals in direct or indirect contact with a pig farm [2]. A new genetic lineage of CA-MRSA for which pigs and other livestock act as a reservoir (LA-MRSA) has been identified. ST398 in pigs was first reported by Armand-Lefèvre et al [3], who found both methicillin-susceptible *S. aureus* (MSSA) and MRSA in pigs and humans [4].

Studies have also confirmed that people in contact with pigs and veal calves are the most likely to be carriers of MRSA-ST398 [5, 6]. Voss et al observed that pig farming is a significant risk factor for MRSA in humans [7]. A subsequent study demonstrated transmission of MRSA-ST398 between pigs and humans [8]. Serious human infections with MRSA-ST398 as an etiologic agent have also been identified in Europe, Asia, Oceania and America [9–15].

In Catalonia (Spain), no data of any kind are available on the prevalence of MRSA-ST398 in the exposed population. Osona, an area of Barcelona province (Catalonia, northeast Spain), is a county with a high density of pig fattening farms and high employment in the sector. According to the latest data, in 2014 there were 674 pig farms in Osona, of which 416 were fattening farms. These data confirm that the county’s capacity to house pigs is very high; in 2014, the density was 904 pigs/km^2^. The swine population, including breeding sows older than 6 months and fattening pigs, was 1.126.446 pigs [16].

The objective of this study was to analyze the prevalence of nasal colonization by MRSA-ST398 in pig farm workers in an area with a high pig population, as well as the prevalence of MRSA-ST398 in pigs and workers from selected farrow-to-finish and fattening farms.

## Methods

We conducted a cross-sectional prevalence study in the county of Osona (Barcelona province, Catalonia, Northeast Spain) from June 2014 to June 2015.

### Selection of farms, workers, and pigs

The study was presented to Osona County farm owners through their business association (Technical Association of Swine Health), assuring them that data confidentiality would be protected. The research was approved by the ethics committee on animal care and use of the Osona regional office of the Agriculture, Livestock, Fisheries, Food and Natural Environment Department of the Catalan government, in accordance with European legislation on animal care, specifically the guidelines related to pig farms [17]. The project was also approved by the research ethics committee of our institution, the Hospital Universitari de Vic.

#### Participating Workers

We studied all the pig farm workers from a total of 83 farms. All workers over the age of 18 who were present at the time of the visit were invited to participate in the study, and all participants signed informed consent forms. We collected epidemiological data on the study participants (age, sex, nationality, years worked on farms) and their medical history (previous hospitalizations and other contact with the health care system).

#### Pigs and Farms

Out of the 83 farms where colonization in pig farmers was studied, 20 of them were selected for pig analysis for MRSA-ST398 colonization, 9 of them fattening (1–6 months) and 11 farrow-to-finish (6 months to 6 years). Twenty farms were selected taking into account the size of the pig population in different county areas. From an area with more than 100,000 pigs, 4 farms were selected, from the areas with between 50,000 and 99,999 pigs, 5 were, in the areas with between 10,000 and 49,999 pigs, 9 were, and in the area with between 1000 and 9999 pigs, 2 farms were. All 20 farms had between 180 and 10.000 pigs. Farms were selected by a simple randomization. Ten pigs per farm were analyzed and one smear was carried out on each pig. At each farm, samples were taken from one or two pigs in each pen, until the proportional number of samples had been collected.

### Collection and processing of samples

A nasal swab was collected from both nostrils of each of the selected pigs and from both nostrils of each participating farm worker. The veterinarian used a hook to take hold of the pig’s snout, immobilized the animal, and collected the sample. After collecting the sample, the hook was removed to release the animal.

All sampling was done with cotton-tipped swabs that were placed in Stuart swab PS+ Viscose (Deltalab, Rubí, Spain). Swabs were stored at 4 °C and transported directly to the laboratory in the Microbiology Department of Hospital Universitari de Vic (Barcelona) for testing.

### MRSA isolation and characterization

The samples were cultured onto a chromogenic MRSA-Brilliance agar (Oxoid, PO5196A, UK), and the results were read after 48 h. Suspected colonies (green) were plated onto blood agar (Oxoid,CM0055,UK), and organism identification was performed using an automated system (bioMérieux Vitek 2). For the confirmation of MRSA isolates, susceptibility for oxacillin and cefoxitin was determined by a disk diffusion test [18], and the presence of the PBP2a protein was analyzed by a latex agglutination test with specific anti-PBP2 monoclonal antibodies (Slidex® MRSA detection- Biomerieux). MRSA strains showed resistance to oxacillin and cefoxitin and were positive for PBP2a protein in the agglutination test.

#### Antibiotics susceptibility testing

Susceptibility testing was carried out by the disk-diffusion method following the Clinical and Laboratory Standards Institute (CLSI) recommendations [18]. The antibiotics tested were as follows: penicillin (10 units), oxacillin (1 μg), erythromycin (15 μg), clindamycin (2 μg), gentamicin (10 μg), rifampicin (5 μg), tetracycline (30 μg), tobramycin (10 μg) trimethoprim sulfamethoxazole (1.25/23.75 μg), ciprofloxacin (5 μg), linezolid (30 μg), and mupirocin (200 μg). Vancomycin and daptomycin susceptibility was studied by broth microdilution. CLSI breakpoints were used for antibiotic susceptibility categorization [18].

Isolates were maintained at -80 °C for other determinations.

### Molecular Typing

#### Multilocus sequence typing (MLST)

All the isolates were analyzed by multilocus sequence typing (MLST), in accordance with the guidelines of the MLST database (http://saureus.mlst.net/).

##### *spa* typing

All MRSA strains were characterized by *spa* typing, performed as previously described [19] using the Ridom StaphType software, version 1.4 (Ridom GmbH Münster, Germany). The *spa* types were assigned according to the Ridom web server (http://www.spaserver.ridom.de/).

### Statistical analysis of the data

Statistical analysis was performed using SPSS 21.0 software.

Categorical variables were expressed as frequency (%) and continuous variables as mean ± standard deviation (SD). Variables not normally distributed (verified by QQ Plot and Kolmogorov-Smirnov) were expressed as median (interquartile range).

The prevalence of positive results in MRSA was estimated with a 95% confidence interval (CI). Statistical significance for intergroup differences was assessed by Pearson’s chi-square or Fisher’s exact test for categorical variables and the Student’s *t* test or Mann-Whitney *U* test for continuous variables, depending on the distribution of the variable. A receiver characteristic operator curve (ROC) was configured in order to calculate a cut-off point with best sensitivity and specificity for the number of pigs on each farm to be associated with the positive results of the pig farm worker. A *p*-value lower than 0.05 was considered statistically significant.

## Results

### Prevalence in pig farm workers and epidemiological characteristics

**Fig. 1:**
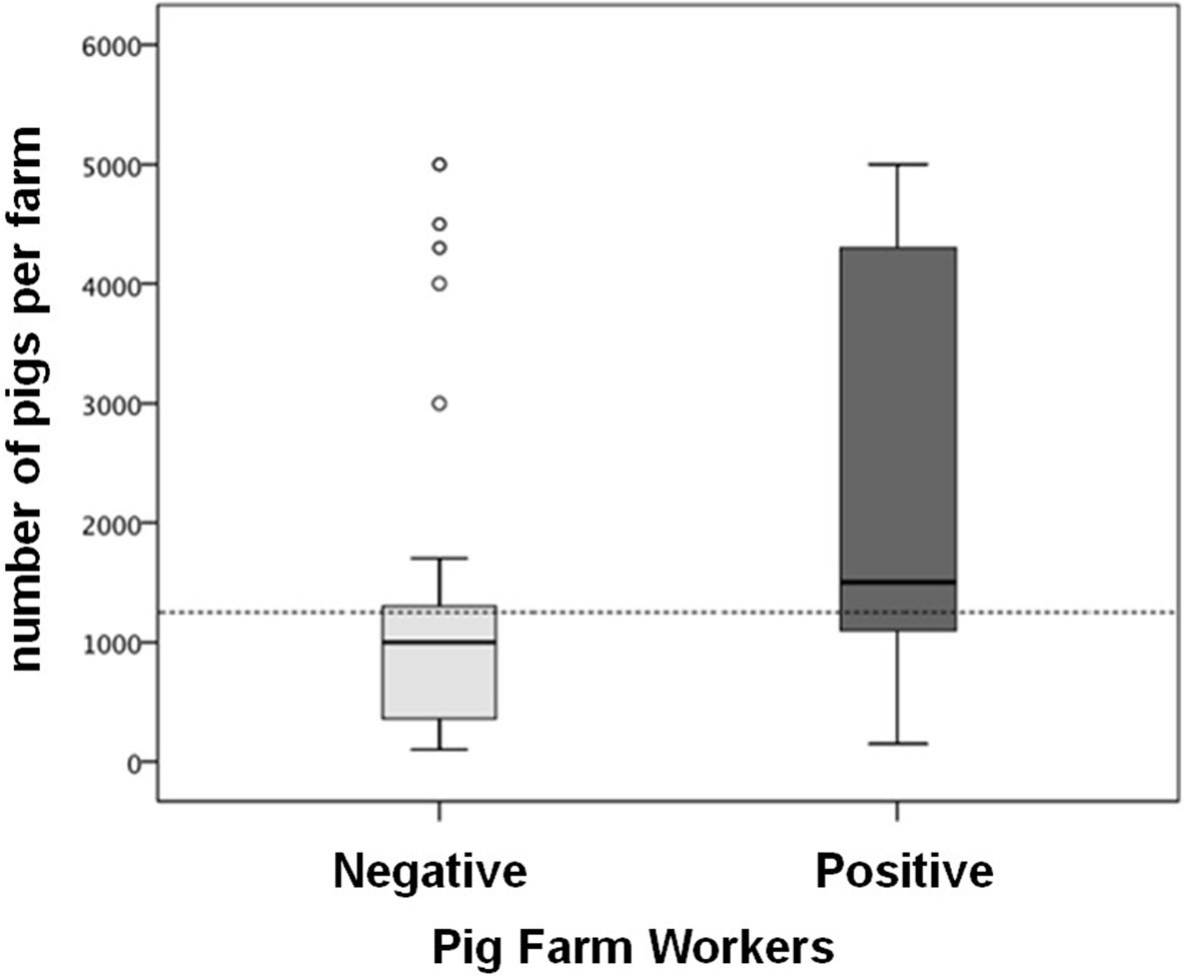
Relationship between MRSA ST398 positive pig farm workers and the number of pigs per farm

One hundred and forty workers from 83 pig farms were studied, with 57.9% (95% CI: 50.0–66.4%) testing positive (81/140) for MRSA, all of them typed as MRSA-ST398. The mean age of the pig farmers was 44.9 ± 13.8 (range:19–80), and most of them were men (94.3%). Only one of the farmers was an immigrant (from Gambia); none had been hospitalized or had taken antibiotics in the last year before the study. With nine exceptions, the participants had no history of disease. The mean number of years worked on farms was 17.5 ± 12.6 (range: 1–50), without significant differences between MRSA-negative and MRSA-positive subjects (17.1 ± 12.5 vs. 17.8 ± 12.7, *p* = 0.763) (Table 1). There was a higher number of pigs in farms where workers tested positive for MRSA-ST398 (1500 [1050–4300] vs. 1000 [300–1300], *p* < 0.001) (Fig. 1). A cut-off point of 1250 pigs per farm was found to be the best discriminator between farms with workers testing positive and negative results in MRSA-ST398. Thus, the percentage of pig farmers working on farms with more than 1250 pigs who tested positive for MRSA ST398 was significantly higher than those working on farms with less than 1250 pigs (75.8 vs. 41.9%, *p* < 0.001).

**Table 1:** Epidemiological characteristics of pig farm workers included in the study

		MRSA ST398		Statistical test and *p* value*
	All	Positive	Negative	
Pig farm workers [n, (%)]	140	81 (57.9)	59 (42.1)	na
Age in years [mean, (SD)]	44.9 (13.8)	44.4 (13.2)	45.5 (14.7)	*p* = 0.631**
Men [n, (%)]	132 (94.3)	75(92.6)	57 (96.6)	*p* = 0.467***
Years of work [mean, (SD)]	17.5(12.6)	17.8 (12.7)	17.1(12.5)	*p* = 0.763**
Medical History[n, (%)]	9 (6)	5 (6.2)	4 (6.8)	*p* = 1.000***
Antibiotics treatment last year %	0	0	0	na
Hospital admission last year %	0	0	0	na
N pigs	1200 (700–3000)	1500 (1050–4300)	1000 (300–1300)	*p* < 0.001****
Fattening farm workers	69 (49.3)	38 (46.9)	31(52.5)	*p* = 0.511*****
farrow-to-finish farm workers	71(51.7)	43(53.1)	28(47.5)	

*na* not applicable

*Level of significance is set at *p* < 0.05

**Student *t*

***Fisher’s exact test

****Mann-Whitney U

*****Pearson’s chi-square

Eight different *spa* types were detected among the 81 MRSA-ST398 isolates obtained from pig farm workers (one/positive farmer): t011 (68%), t108 (6%), t034 (6%), t1451 (7%), t1197 (5%), t1456 (4%), t2346 (3%) and t943 (1%). Table 2 shows the correlation of the phenotypes of antibiotic resistance with *spa* types in isolates of pig farmers who were MRSA-ST398 positive, and also the number of farms in which each *spa*-type was detected.

**Table 2:** Characteristics of the 81 MRSA^a^ ST398 isolates detected in the 140 pig farmers (PF) from the 83 farms tested

*spa* types of isolates	Number of isolates	Number of positive farms/	% of resistance for non-beta-lactam antibiotics^b^						
		number of positive PFs	TET	ERY	CLI	CIP	GEN	TOB	SXT
t011	55	13/55	100	90.9	100	34.5	27.2	18.1	25.4
t1456	3	3/3	100	100	100	100	0	0	0
t1451	6	6/6	100	100	100	100	0	33.3	16.6
t108	5	4/5	100	100	100	60	0	20	60
t943	1	1/1	100	100	100	100	0	0	0
t1197	4	3/4	100	75	100	100	25	25	75
t034	5	4/5	100	80	100	60	0	60	40
t2346	2	2/2	100	100	100	0	0	0	0

^a^All MRSA isolates showed susceptibility to vancomycin, daptomycin, linezolid, mupirocin and rifampicin

^b^Antimicrobials: TET, tetracycline; ERY, erythromycin; CLI, clindamycin; GEN, gentamicin; CIP, ciprofloxacin; SXT, trimethoprim-sulfamethoxazole; TOB: tobramycin

### Prevalence in pigs and correlation with isolates of farmers

Twenty of the 83 pig farms were selected for the analysis of MRSA-ST398 colonization in pigs and to determine the correlation with the results in the corresponding farmers. For this purpose, nasal swabs from 200 pigs of these 20 farms were collected (101 fattening pigs and 99 sows in service units) and analyzed. Ninety-two of the animals (46% (IC 95%: 39–53%)) were MRSA-positive, all of them of the genetic lineage ST398 (41.4% in fattening pigs and 50.5% in sows, *p* = 0.198). The percentage of positive results in fattening farms was similar to the percentage of positive results in farrow-to-finish farms (43.3 vs. 48.2%, *p* = 0.494). The median of pigs per farm was 1.525 (interquartile range: 1.125–4.500) with a minimum of 180 and a maximum of 10.000 pigs. Of the 20 pig farms selected for the analysis of both farmers and pigs, workers colonized with MRSA-ST398 were identified in all of them.

The MRSA-ST398 strains recovered from pigs presented 7 different *spa* types: t011 (71%), t034 (8%), t2346 (2%), t108 (11%), t1197 (1%), t1456 (6%), and t1451 (1%). Table 3 shows the results of the MRSA-ST398 isolates of pigs and farmers from the 20 farms, including the distribution of *spa*-types and the phenotypes of antibiotic resistance. In most of the farms (19/20), it was possible to detect MRSA-ST398 with a similar *spa*-type and phenotype of antibiotic resistance in pigs and farmers (Table 3). Most of the MRSA-ST398 strains presented a multi-resistant phenotype (resistance to at least three different families of antibiotics), including in all cases tetracycline resistance. All MRSA isolates showed resistance for tetracycline and susceptibility for vancomycin, daptomycin, linezolid, mupirocin and rifampicin.

**Table 3:** Frequency of detection of MRSA ST398 isolates in pig farmers and pigs in the 20 tested farms and genetic lineages (MLST and *spa*-type) and antimicrobial resistance phenotypes of recovered isolates

	Pig Farmers			Pigs			Antimicrobial resistance
Farm/number of pigs	Positive/total	MLST	*spa* type	Positive/total	MLST	*spa* type	Phenotype of resistance for non-beta-lactams^a^
(*)Farm 1/4500	3/3	ST 398	t011	3/10	ST 398	t011	TET,GEN,TOB,CLI,ERY,SXT
				1/10	ST 398	t011	TET,GEN,TOB,CLI,ERY
(*)Farm 2/1200	1/4	ST 398	t011	1/10	ST 398	t011	TET, TOB, CLI, ERY,CIP,SXT
	1/4	ST 398	t011	3/10	ST 398	t011	TET, CIP, SXT
(**)Farm 3/180	1/1	ST 398	t034	5/10	ST 398	t034	TET, TOB, CLI, CIP
(*)Farm 4/800	2/2	ST 398	t011	4/10	ST 398	t011	TET,CLI,ERY
(*)Farm 5/1500	1/1	ST 398	t011	4/10	ST 398	t011	TET,CLI,ERY,SXT
(**)Farm 6/2000	1/1	ST 398	t011	4/10	ST 398	t011	TET,CLI,ERY,CIP
(*)Farm 7/1700	3/8	ST 398	t011	1/10	ST 398	t011	TET,CLI,ERY,CIP
	1/8	ST 398	t011	1/10	ST 398	t011	TET,CLI,ERY,GEN,TOB,SXT
	2/8	ST 398	t034	2/10	ST 398	t034	TET,CLI,ERY,CIP,TOB,SXT
	1/8	ST 398	t1451				TET,CLI,ERY,CIP,TOB,SXT
(*)Farm 8/1300	1/2	ST 398	t2346	2/10	ST 398	t2346	TET,CLI,ERY
	1/2	ST 398	t108	2/10	ST 398	t108	TET,CLI,ERY,TOB,SXT
(*)Farm 9/340	1/1	ST 398	t011	7/10	ST 398	t011	TET,CLI,ERY
(**)Farm 10/5000	1/3	ST 398	t011	3/10	ST 398	t011	TET,CLI,ERY,CIP,TOB, SXT
	1/3	ST 398	t1197	1/10	ST 398	t1197	TET,CLI,ERY,CIP,SXT
(*)Farm 11/2700	1/1	ST 398	t011	4/10	ST 398	t011	TET,CLI,ERY
(**)Farm 12/1550	1/1	ST 398	t011	5/10	ST 398	t011	TET,CLI,ERY,CIP,TOB,SXT
(*)Farm 13/5000	1/1	ST 398	t1197	1/10	ST 398	t1197	TET,CLI,CIP,TOB,
				3/10	ST 398	t1456	TET,CLI,ERY
(**)Farm 14/5000	1/1	ST 398	t1451	1/10	ST 398	t1451	TET,CLI,ERY,CIP,TOB
				2/10	ST 398	t011	TET,CLI,ERY,CIP
				1/10	ST 398	t1456	TET,CLI,ERY
(**)Farm 15/600	1/1	ST 398	t011	4/10	ST 398	t011	TET,CLI,ERY,CIP,SXT
(**)Farm 16/1200	1/1	ST 398	t108				TET,CLI,ERY,CIP
				4/10	ST 398	t011	TET,CLI,ERY,CIP,TOB
(*)Farm 17/10000	2/5	ST 398	t011				TET,CLI,ERY,CIP,SXT
	1/5	ST 398	t1451				TET,CLI,ERY,CIP,
	2/5	ST 398	t108	8/10	ST 398	t108	TET,CLI,ERY,TOB,SXT
(**)Farm 18/1300	2/4	ST 398	t011	2/10	ST 398	t011	TET,CLI,ERY,CIP
	1/4	ST 398	t011	1/10	ST 398	t011	TET,CLI,ERY
	1/4	ST 398	t011	2/10	ST 398	t011	TET,CLI,ERY,CIP,SXT
(**)Farm 19/1500	1/4	ST 398	t011	1/10	ST 398	t011	TET,CLI,ERY,CIP
	1/4	ST 398	t011	2/10	ST 398	t011	TET,CLI,ERY,CIP,GEN,TOB,SXT
	1/4	ST 398	t1451	1/10	ST 398	t1451	TET,CLI,ERY,CIP
(*)Farm 20/4500	2/4	ST 398	t011	5/10	ST 398	t011	TET,CLI,ERY,GEN,TOB,SXT
	1/4	ST 398	t011	1/10	ST 398	t011	TET,CLI,ERY,GEN,TOB
Total	43/49			92/200			

(*) farrow-to-finish; (**) fattening

^a^Antimicrobials: TET, tetracycline; ERY, erythromycin; CLI, clindamycin; GEN, gentamicin; CIP, ciprofloxacin; SXT, trimethoprim-sulfamethoxazole; TOB: tobramycin

## Discussion

The prevalence of LA-MRSA of the ST398 lineage in pig farm workers in Osona region (Catalonia, Spain) is 58%, considerably higher than the 9% reported in the only previous study of MRSA in livestock farmers in Spain [20], and also much higher than the prevalence of MRSA in the general healthy human population in Spain (<0.5%) [21]. This is the first study of LA-MRSA prevalence in pig farm workers conducted in Catalonia.

In the previous study referred to performed in Spain (Canary Islands), a relatively low prevalence of MRSA (9%) was detected in nasal samples of pig workers (including farmers and workers of slaughterhouses), although higher frequency was found when only pig farm workers were considered (15%) [20]. In our study at least in part, the workers studied were exclusively from pig farms, which could explain the higher prevalence of MRSA-ST398 (58%); other factor could be the higher density of pigs in Osona region compared with Canary Islands (data not shown). Other European studies found a lower prevalence of LA-MRSA in pig farmers. A study in Switzerland were not detected pig farmers with LA-MRSA [22], in another study in a region of Germany the prevalence was 25% [23]. Our findings are close to those of a study in the Netherlands, where 63% of pig farmers were colonized with the ST398 strain [24].

Carrier *status* of LA-MRSA in humans may be related to direct contact with pigs [8] or to human-human transmission [25], and the colonization may be intermittent or persistent [24, 26–29]. Therefore, it seems that the risk factors for persistent MRSA-ST398 carrier *status* depend on the intensity of animal contact [26], together with an age range of 40–49 years, a 40-h working week, and assisting sows with birthing [24]. To a lesser, but still significant extent, lack of hand-washing when leaving the barns was associated with persistent MRSA-positive nasal swabs [24]. Our study shows that the size of the farm could be an important factor for MRSA-ST398 colonization; thus, working on farms with more than 1250 pigs seems to be associated with a higher risk for nasal MRSA-ST398 colonization of farmers; nevertheless, no difference was observed between carriers and non-carriers of MRSA when the number of years worked on the farm was evaluated.

Moreover, in all the farms where both the farmer and the pigs were studied, the results for MRSA-ST398 were positive and the *spa*-type and the resistance phenotype was similar. This finding suggests that humans and animals have interrelated strains. It is known that LA-MRSA ST398 originated as MSSA in humans and exemplifies a bidirectional zoonotic exchange, underscoring the potential public health risks [30].

The prevalence of MRSA found in pigs (46%) was similar in comparison to other European studies. For example, in Belgium, an estimated 44% were carriers[31]; in Germany, a prevalence of 52% was reported for fattening farms [32], and there was 56% prevalence in pig holding companies in the Netherlands [33]. Moreover, in La Rioja (Northern Spain), Gómez-Sanz described a prevalence of 21 and 49% in fattening and suckling pigs, respectively, in this case at the slaughterhouse level [34]. Another study showed that 28% of Iberian pigs were colonized; these animals have little contact with pig farmers [35]. Furthermore, in our study we observed that pig farmers working in farms with pig populations are carriers of MRSA ST398. This finding may suggest that farms with more pigs could be more likely to have MRSA-ST398-positive pig farmers. A recent German study showed that the number of pigs per farm was directly related to the probability of LA-MRSA colonization [36]. Similarly, in The Netherlands, a lower quantity of antibiotics is used on smaller farms, which could help to explain this lower prevalence of LA-MRSA on small farms [37].

The animals studied were sows and fattening pigs, with a slightly higher, although not statistically significant prevalence found in sows. This may be because fattening pigs live for only 6 months, at which point they are sent to the slaughterhouse. As a result, there may not be enough time for these animals to be colonized. Sows, by contrast, live on farms for an average of 5 years. This trend toward a linear relationship between increased colonization by LA-MRSA and a longer lifespan was previously described by Broens et al [38].

All the MRSA identified were of the same genetic lineage (ST398), and the most frequent *spa*-type found in the workers was t011, followed by t108, t1451, t1197, and t1456; in pigs, the most frequent ones were t011, t108 and t1456. This finding agrees with other European studies in Spain, Belgium, Germany and The Netherlands [32, 39–41].

In terms of phenotypes of antibiotic resistance, all our MRSA-ST398 isolates showed tetracycline resistance. Previous studies have demonstrated that tetracycline-resistance is a good phenotypic marker of MRSA of the lineage ST398 [41, 42] and it is known that the *tet*(M) gene, encoding tetracycline resistance, is integrated into the SCC*mec* element in MRSA ST398 strains. The MRSA strains of this study showed high rates of resistance to erythromycin, and even higher for clindamycin. In ten of the pig farmers tested, MRSA isolates with dissociated clindamycin-erythromycin resistance were detected (clindamycin-resistance/erythromycin-susceptibility), probably due to unusual clindamycin resistance genes (as *InuA* or *vga*A), enriched in isolates of this genetic lineage [43, 44]. Most of the MRSA isolates obtained from farmers and pigs in this study showed a wide phenotype of antibiotic resistance, characteristic of this MRSA genetic lineage [45].

One limitation of the study is that other groups of pigs from the farms were not included. To minimize this effect the majority of groups of pigs (fattening pigs and sows in service units) from each farm have been included in the study. We are currently exploring the possibility of conducting a multicenter study in multiple areas of Spain with a high density of pigs. Additionally, there may be a bias in the study related to the age when the nasal swabs were carried out on the pigs: when the samples were taken, the age of the animals was not a selection criterion. On the other hand, all participating farms used the same veterinarian and the same company to monitor sanitation. Therefore, our findings about antibiotic control cannot necessarily be generalized to other farms.

## Conclusions

We describe for the first time MRSA-ST398 prevalence in Catalonia, the region of Spain with the highest density of pigs. The observed prevalence of LA-MRSA in pig farm workers in the county of Osona (Catalonia, Spain) was high. Pig farmers working on farms with more than 1250 pigs are more likely to be LA-MRSA positive. The prevalence of the MRSA found in pigs (46%) was similar in comparison to other European countries. On farms where workers and pigs were studied both were positive.

## Abbreviations

CA-MRSA: Community acquired methicillin-resistant *Staphylococcus aureus*
CI: Confidence interval
HA-MRSA: Hospital acquired methicillin-resistant *Staphylococcus aureus*
LA-MRSA: Livestock acquired methicillin-resistant *Staphylococcus aureus*
MLST: Multilocus sequence typing
MRSA: Methicillin-resistant *Staphylococcus aureus*
MSSA: Methicillin-susceptible *Staphylococcus. aureus*
PF: Pig farmer
PFs: Pig farmers
SD: Standard deviation

## References

[1] Que M, Mandell GL, Bennett JE DR (2010). Staphylococcus aureus. Principles and practice of infectious diseases.

[2] Pan A, Battisti A, Zoncada A, Bernieri F, Boldini M (2009). Community acquired methicillin-resistant *Staphylococcus aureus* ST398 infection, Italy. Emerg Infect Dis.

[3] Armand-Lefevre L, Ruimy R, Andremont A (2005). Clonal comparison of *Staphylococcus aureus* isolates from healthy pig farmers, human controls, and pigs. Emerg Infect Dis.

[4] De Neeling AJ, van den Broek MJM, Spalburg EC, van Santen-Verheuvel MG, Dam-Deisz WDC, Boshuizen HC (2007). High prevalence of methicillin resistant *Staphylococcus aureus* in pigs. Vet Microbiol.

[5] Huijsdens XW, Bosch T, van Santen-Verheuvel MG, Spalburg E, Pluister GN, van Luit M, et al. Molecular characterisation of PFGE non-typable methicillin-resistant *Staphylococcus aureus* in The Netherlands, 2007. Euro Surveill. 2009;14:19335. 10.2807/ese.14.38.19335-en 19814956

[6] Huijsdens XW, van Dijke BJ, Spalburg E, van Santen-Verheuvel MG, Heck MEOC, Pluister GN (2006). Community-acquired MRSA and pig-farming. Ann Clin Microbiol Antimicrob.

[7] Voss A, Loeffen F, Bakker J, Klaassen C, Wulf M. Methicillin-resistant *Staphylococcus aureus* in pig farming. Emerg Infect Dis. 2005;11:1965-6. 10.3201/eid1112.050428 PMC3367632 16485492

[8] van Loo I, Huijsdens X, Tiemersma E, de Neeling A, van de Sande-Bruinsma N, Beaujean D (2007). Emergence of methicillin-resistant *Staphylococcus aureus* of animal origin in humans. Emerg Infect Dis.

[9] Garcia-Graells C, Antoine J, Larsen J, Catry B, Skov R, Denis O (2012). Livestock veterinarians at high risk of acquiring methicillin-resistant *Staphylococcus aureus* ST398. Epidemiol Infect.

[10] Groves MD, O’Sullivan MVN, Brouwers HJM, Chapman TA, Abraham S, Trott DJ (2014). *Staphylococcus aureus* ST398 detected in pigs in Australia. J Antimicrob Chemother.

[11] Horgan M, Abbott Y, Lawlor PG, Rossney A, Coffey A, Fitzgerald GF (2011). A study of the prevalence of methicillin-resistant *Staphylococcus aureus* in pigs and in personnel involved in the pig industry in Ireland. Vet J.

[12] Lozano C, Aspiroz C, Ezpeleta AI, Gómez-Sanz E, Zarazaga M, Torres C (2011). Empyema caused by MRSA ST398 with atypical resistance profile, Spain. Emerg Infect Dis.

[13] Smith TC, Male MJ, Harper AL, Kroeger JS, Tinkler GP, Moritz ED (2009). Methicillin-resistant *Staphylococcus aureus* (MRSA) strain ST398 is present in midwestern U.S. swine and swine workers. PLoS One.

[14] Witte W, Strommenger B, Stanek C, Cuny C (2007). Methicillin-resistant *Staphylococcus aureus* ST398 in humans and animals, Central Europe. Emerg Infect Dis.

[15] Yan X, Yu X, Tao X, Zhang J, Zhang B, Dong R (2014). *Staphylococcus aureus* ST398 from slaughter pigs in northeast China. Int J Med Microbiol.

[16] Idescat (Institut D’EStadística de CATalunya): Anuari estadístic de Catalunya. Agricultura, ramaderia i pesca. Ramaderia. Explotacions ramaderes. Per espècies. Comarques, àmbits i provincies. Explotacions. Catalunya: Departament d’Agricultura, Ramaderia, Pesca, Alimentació i Medi natural. http://www.idescat.cat/pub/aec/454 (2014). Accessed 15 Nov 2015.

[17] Official Journal of the European Union: Directiva 2008/120/CE del consejo de 18 de diciembre de 2008 relativa a las normas mínimas para la protección de cerdos. http://eurlex.europa.eu/LexUriServ/LexUriServ.do?uri=OJ:L:2009:047:0005:0013:ES:PDF (2009). Accessed 15 Jan 2016.

[18] CLSI (2014). Performance standards for antimicrobial susceptibility testing; Twenty-Fourth Informational Supplement. CLSI document M100-S24.

[19] Shopsin B, Gomez M, Montgomery SO, Smith DH, Waddington M, Dodge DE (1999). Evaluation of protein a gene polymorphic region DNA sequencing for typing of *staphylococcus aureus* strains. J Clin Microbiol.

[20] Morcillo A, Castro B, Rodríguez-Álvarez C, González JC, Sierra A, Montesinos MI (2012). Prevalence and characteristics of methicillin-resistant *Staphylococcus aureus* in pigs and pig workers in Tenerife, Spain. Foodborne Pathog Dis.

[21] Lozano C, Gómez-Sanz E, Benito D, Aspiroz C, Zarazaga M, Torres C (2011). *Staphylococcus aureus* nasal carriage, virulence traits, antibiotic resistance mechanisms, and genetic lineages in healthy humans in Spain, with detection of CC398 and CC97 strains. Int J Med Microbiol.

[22] Huber H, Koller S, Giezendanner N, Stephan R, Zweifel C (2010). Prevalence and characteristics of meticillin-resistant *Staphylococcus aureus* in humans in contact with farm animals, in livestock, and in food of animal origin, Switzerland, 2009. Euro Surveill.

[23] Dahms C, Hübner N-O, Cuny C, Kramer A (2014). Occurrence of methicillin-resistant *Staphylococcus aureus* in farm workers and the livestock environment in Mecklenburg-Western Pomerania, Germany. Acta Vet Scand.

[24] van Cleef BAGL, van Benthem BHB, Verkade EJM, van Rijen M, Kluytmans-van den Bergh MFQ, Schouls LM (2014). Dynamics of methicillin-resistant *Staphylococcus aureus* and methicillin-susceptible *Staphylococcus aureus* carriage in pig farmers: a prospective cohort study. Clin Microbiol Infect.

[25] McCarthy AJ, van Wamel W, Vandendriessche S, Larsen J, Denis O, Garcia-Graells C (2012). *Staphylococcus aureus* CC398 clade associated with human-to-human transmission. Appl Environ Microbiol.

[26] Graveland H, Wagenaar JA, Bergs K, Heesterbeek H, Heederik D (2011). Persistence of livestock associated MRSA CC398 in humans is dependent on intensity of animal contact. PLoS One.

[27] Köck R, Loth B, Köksal M, Schulte-Wülwer J, Harlizius J, Friedrich AW (2012). Persistence of nasal colonization with livestock-associated methicillin-resistant *Staphylococcus aureus* in pig farmers after holidays from pig exposure. Appl Environ Microbiol.

[28] van Cleef BAGL, Graveland H, Haenen APJ, van de Giessen AW, Heederik D, Wagenaar JA (2011). Persistence of livestock-associated methicillin-resistant *Staphylococcus aureus* in field workers after short-term occupational exposure to pigs and veal calves. J Clin Microbiol.

[29] Verkade E, van Benthem B, den Bergh MK, van Cleef B, van Rijen M, Bosch T (2013). Dynamics and determinants of *Staphylococcus aureus* carriage in livestock veterinarians: a prospective cohort study. Clin Infect Dis.

[30] Price LB, Stegger M, Hasman H, Aziz M, Larsen J, Andersen PS (2012). *Staphylococcus aureus* CC398: host adaptation and emergence of methicillin resistance in livestock. MBio.

[31] Crombé F, Willems G, Dispas M, Hallin M, Denis O, Suetens C (2012). Prevalence and antimicrobial susceptibility of methicillin-resistant *Staphylococcus aureus* among pigs in Belgium. Microb Drug Resist.

[32] Alt K, Fetsch A, Schroeter A, Guerra B, Hammerl JA, Hertwig S (2011). Factors associated with the occurrence of MRSA CC398 in herds of fattening pigs in Germany. BMC Vet Res.

[33] Broens EM, Graat EAM, van der Wolf PJ, van de Giessen AW, van Duijkeren E, Wagenaar JA (2011). MRSA CC398 in the pig production chain. Prev Vet Med.

[34] Gómez-Sanz E, Torres C, Lozano C, Fernández-Pérez R, Aspiroz C, Ruiz-Larrea F (2010). Detection, molecular characterization, and clonal diversity of methicillin-resistant *Staphylococcus aureus* CC398 and CC97 in Spanish slaughter pigs of different age groups. Foodborne Pathog Dis.

[35] Porrero MC, Wassenaar TM, Gómez-Barrero S, García M, Bárcena C, Alvarez J (2012). Detection of methicillin-resistant *Staphylococcus aureus* in Iberian pigs. Lett Appl Microbiol.

[36] Fromm S, Beißwanger E, Käsbohrer A, Tenhagen B-A (2014). Risk factors for MRSA in fattening pig herds - A meta-analysis using pooled data. Prev Vet Med.

[37] Mevius DJ, Wit B, van Pelt WBN. Usage of antibiotics in animal husbandry in the Netherlands. In: Central Veterinary Institute of Wageningen UR, editor. Monitoring of Antimicrobial Resistance and Antibiotic Usage in Animals in the Netherlands in 2009. 2009th ed. Lelystad: MARAN-2009; 2009.

[38] Broens EM, Espinosa-Gongora C, Graat E a M, Vendrig N, Van Der Wolf PJ, Guardabassi L (2012). Longitudinal study on transmission of MRSA CC398 within pig herds. BMC Vet Res.

[39] Bosch T, de Neeling AJ, Schouls LM, van der Zwaluw KW, Kluytmans JAJW, Grundmann H (2010). PFGE diversity within the methicillin-resistant *Staphylococcus aureus* clonal lineage ST398. BMC Microbiol.

[40] Brandt KM, Mellmann A, Ballhausen B, Jenke C, van der Wolf PJ, Broens EM (2013). Evaluation of multiple-locus variable number of tandem repeats analysis for typing livestock-associated methicillin-resistant *Staphylococcus aureus*. PLoS One.

[41] Camoez M, Sierra JM, Pujol M, Hornero A, Martin R, Domínguez MA (2013). Prevalence and molecular characterization of methicillin-resistant *Staphylococcus aureus* ST398 resistant to tetracycline at a Spanish hospital over 12 years. PLoS One.

[42] Lozano C, Rezusta A, Gómez P, Gómez-Sanz E, Báez N, Martin-Saco G (2012). High prevalence of spa types associated with the clonal lineage CC398 among tetracycline-resistant *Staphylococcus aureus* strains in a Spanish hospital. J Antimicrob Chemother.

[43] Lozano C, Aspiroz C, Rezusta A, Gómez-Sanz E, Simón E, Gómez P, Ortega C, Revillo MJ, Zarazaga M, Torres C (2012). Identification of novel *vga*(A)-carrying plasmids and a Tn*5406*-like transposon in meticillin-resistant *Staphylococcus aureus* and *Staphylococcus epidermidis* of human and animal origin. Int J Antimicrob Agents.

[44] Lozano C, Aspiroz C, Sáenz Y, Ruiz-García M, Royo-García G, Gómez-Sanz E, Ruiz-Larrea F, Zarazaga M, Torres C (2012). Genetic environment and location of the *lnu*(A) and *lnu*(B) genes in methicillin resistant *Staphylococcus aureus* and other staphylococci of animal and human origin. J Antimicrob Chemother.

[45] Benito D, Lozano C, Rezusta A, Ferrer I, Vasquez MA, Ceballos S (2014). Characterization of tetracycline and methicillin resistant *Staphylococcus aureus* strains in a Spanish hospital: Is livestock-contact a risk factor in infections caused by MRSA CC398?. Int J Med Microbiol.

